# Supplementary material for: Comparing machine learning with case-control models to identify confirmed dengue cases
Source: PLoS Negl Trop Dis. 2020 Nov 10;14(11):e0008843. doi: 10.1371/journal.pntd.0008843 (PMC7654779; doi:10.1371/journal.pntd.0008843)

Supporting Information

S1 Fig Epidemic curve of the 2015 dengue outbreak in Tainan city and monthly case distribution trend in current study.

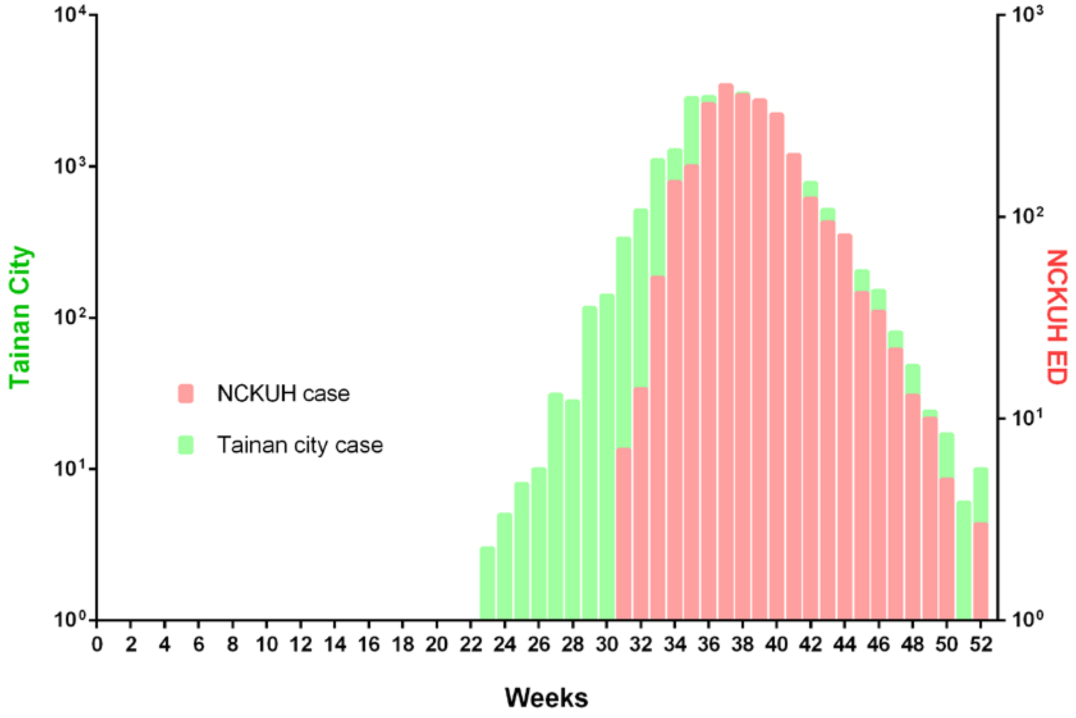

Supplement: S1 Fig — (PDF) [file pntd.0008843.s001.pdf]
