# Supplementary material for: Comparing machine learning with case-control models to identify confirmed dengue cases
Source: PLoS Negl Trop Dis. 2020 Nov 10;14(11):e0008843. doi: 10.1371/journal.pntd.0008843 (PMC7654779; doi:10.1371/journal.pntd.0008843)

S2 Fig A decision tree generated with prior set to 0.388. This particular tree produced 90.1% sensitivity but only 63.6% specificity. The prediction algorithm traverses the decision tree starting from the root, which is the node at the top of the tree. Each of the branches originating from a node is associated with a criterion of the attribute values. The prediction algorithm moves down along the tree based on the attribute values of the subject for which a prediction is to be made. The "n+" and "n-" symbols in each node respectively denote the number of positive subjects and the number of negative subjects in the training dataset that meet the criteria specified along the path from the root to this particular node. If n+ in a node is larger than n-, then the node is colored by red. Otherwise, the node is colored by blue.

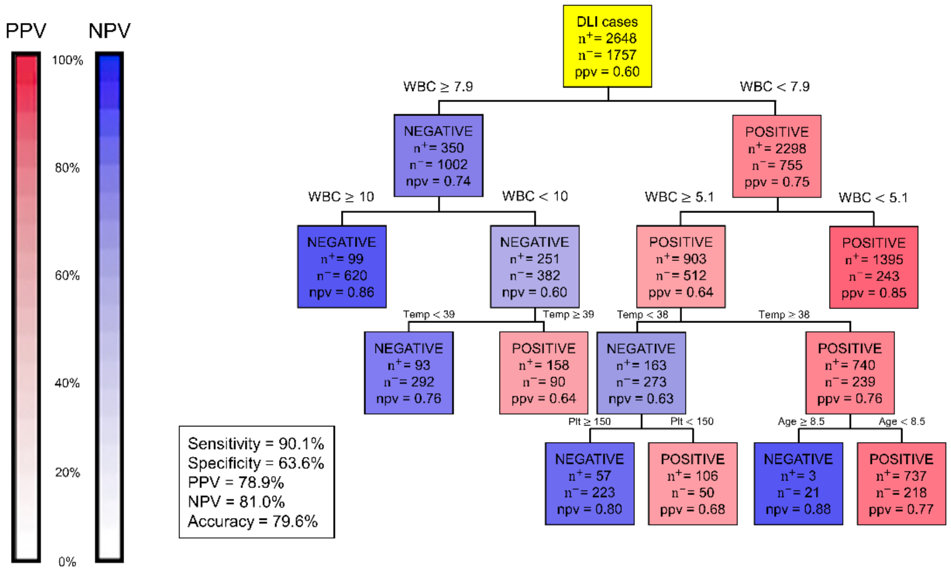

Supplement: S2 Fig — This particular tree produced 90.1% sensitivity but only 63.6% specificity. The prediction algorithm traverses the decision tree starting from the root, which is the node at the top of the tree. Each of the branches originating from a node is associated with a criterion of the attribute values. The prediction algorithm moves down along the tree based on the attribute values of the subject for which a prediction is to be made. The “n+” and “n-” symbols in each node respectively denote the number of positive subjects and the number of negative subjects in the training dataset that meet the criteria specified along the path from the root to this particular node. If n+ in a node is larger than n-, then the node is colored by red. Otherwise, the node is colored by blue. (PDF) [file pntd.0008843.s002.pdf]
