# Supplementary material for: Comparing machine learning with case-control models to identify confirmed dengue cases
Source: PLoS Negl Trop Dis. 2020 Nov 10;14(11):e0008843. doi: 10.1371/journal.pntd.0008843 (PMC7654779; doi:10.1371/journal.pntd.0008843)
Supplement: S1 Table — Pre-peak: Before Epidemic Peak in the Epidemic Curve; SD: Standard Deviation ICU: Intensive Care Units; BP: Blood Pressure; BPM: Heart Rate as Beats per Minute, WBCs: White Blood Cells; CVA: cerebral vascular accident CKD: Chronic Kidney Disease, DM: Diabetes Mellitus. (PDF) [file pntd.0008843.s004.pdf]

S1 Table. Comparison of the excluded patients and included cases of ED patients at NCKU Hospital, Jan. 1 to Dec. 31, 2015 in this study.

		Excluded Patients	Included Cases	p-value*
**Emergency Visits**		**1474**	**4894**	
**Age-Group**	0-17 years	161 (10.92%)	357 (7.29%)	<0.001
	18-64 years	935 (63.43%)	3253 (66.47%)	
	≥65 years	378 (25.64%)	1284 (26.24%)	
**Age (mean ± SD)**		45.80 ± 22.58	46.82 ± 22.54	0.1258
**Gender**	Male	691 (46.88%)	2476 (50.59%)	0.0135
	Female	783 (53.12%)	2418 (49.41%)	
**Severity**				
Non-Hospitalized		1148 (77.88%)	3938 (80.47%)	0.1285
Hospitalized		270 (18.32%)	812 (16.59%)	
ICU		24 (1.63%)	65 (1.33%)	
Death		32 (2.17%)	79 (1.61%)	
**Triage Vital Signs**		**(mean±SD)**	**(mean±SD)**	
Body Temperature(°C)		37.64±1.43	38.18±1.00	<0.001
Systolic BP (mmHg)		127±21	134±22	<0.001
Diastolic BP (mmHg)		79±14	82±15	<0.001
Heart Rate (BPM)		92±20	101±20	<0.001
Respiratory Rate (/min)		20±2	20±3	0.0240
**Blood Counts**		**(mean±SD)**	**(mean±SD)**	
WBC ($10^3/\mu L$)		4.81±3.33	6.81±3.93	<0.001
Platelet ($10^3/\mu L$)		114.63±79.60	171.10±75.75	<0.001
Hemoglobin (g/dL)		13.57±1.93	13.24±1.87	<0.001
**Comorbidities**		-	-	
Heart diseases		153 (10.38%)	545 (11.14%)	0.4153
Cerebrovascular disease		55 (3.73%)	265(5.41%)	0.0095
Chronic kidney disease		288 (19.54%)	1089 (22.25%)	0.0265
Cirrhosis of liver		144 (9.77%)	435 (8.89%)	0.3024
DM		253 (17.16%)	880 (17.98%)	0.4721
Hypertension		264 (17.91%)	938 (19.17%)	0.2801
Cancer		208 (14.11%)	926 (18.92%)	<0.001

Pre-peak: Before Epidemic Peak in the Epidemic Curve; **SD:** Standard Deviation
ICU: Intensive Care Units; **BP:** Blood Pressure; **BPM:** Heart Rate as Beats per Minute,
WBCs: White Blood Cells; **CVA:** cerebral vascular accident
CKD: Chronic Kidney Disease, **DM:** Diabetes Mellitus
