# Supplementary material for: Comparing machine learning with case-control models to identify confirmed dengue cases
Source: PLoS Negl Trop Dis. 2020 Nov 10;14(11):e0008843. doi: 10.1371/journal.pntd.0008843 (PMC7654779; doi:10.1371/journal.pntd.0008843)
Supplement: S2 Table — (PDF) [file pntd.0008843.s005.pdf]

S2 Table. Cut-offs employed to stratify numerical variables for building prediction models

Variable Symbols	Categorical Groups	Cut-off Values
**Gender**	Female	
	Male	
**Body Temperature (Temp)**	Normal	Body Temperature < 38℃
	Fever	Body Temperature ≥ 38℃
**Age**	Adult (Reference)	18 ≤ Age < 65 years
	Young	Age < 18 years
	Elder	Age ≥ 65years
**Glasgow Coma Scale (GCS)_total**	Normal	GCS_total=15
	Abnormal	GCS_total<=14
**Platelets (PLTs)**	Normal	Plt count ≥ 100 ($10^3/\mu L$)
	Low	Plt count < 100 ($10^3/\mu L$)
**Hemoglobin (Hb)**	Normal	Female normal range : 11.6~14<br>Male normal range : 13.5~17
	Low	
	High	
**White Blood Cells (WBCs)**	Normal	3.2 ($10^3/\mu L$) < WBC count < 10 ($10^3/\mu L$)
	Low	WBC count ≤ 3.2 ($10^3/\mu L$)
	High	WBC count ≥ 10 ($10^3/\mu L$)
**Systolic Blood Pressure (SBP)**	Low	SBP < 100(mmHg)
	Normal	100 ≤ SBP ≤ 140 (mmHg)
	High	140 < SBP (mmHg)
**Diastolic Blood Pressure (DBP)**	Low	DBP < 70 (mmHg)
	Normal	70 ≤ DBP ≤ 90 (mmHg)
	High	90 < DBP (mmHg)
**Heart Rate**	Low	Pulse <6 0 (BPM)
	Normal	60 ≤ Pulse≤ 100 (BPM)
	High	100 < Pulse(BPM)
**Respiratory Rate**	Low	Breath < 12 (/min)
	Normal	12 ≤ Breath ≤ 18 (/min)
	High	18 < Breath (/min)
