# Supplementary material for: Comparing machine learning with case-control models to identify confirmed dengue cases
Source: PLoS Negl Trop Dis. 2020 Nov 10;14(11):e0008843. doi: 10.1371/journal.pntd.0008843 (PMC7654779; doi:10.1371/journal.pntd.0008843)
Supplement: S3 Table — SBP: Systolic Blood Pressure; DBP: Diastolic Blood Pressure, WBC: White Blood Cells; GCS: Glasgow Coma Scale, CVA: cerebral vascular accident; CKD: Chronic Kidney Disease, DM: Diabetes Mellitus. (PDF) [file pntd.0008843.s006.pdf]

S3 Table. Crude Odds Ratios with 95% Confidence Intervals in parentheses

**Input Variables**	**Crude Odds Ratios**	**95% Confidence Intervals (C.I).**
Fever	1.92	(1.70, 2.16)
Male vs female	0.94	(0.84, 1.05)
Elder vs adult	1.71	(1.49, 1.97)
Young vs Adult	0.70	(0.56, 0.87)
Low Platelets (PLTs)	3.95	(3.23, 4.84)
High Hemoglobin (Hb)	1.13	(0.90, 1.41)
Low Hb	0.67	(0.59, 0.76)
High WBC	0.10	(0.08, 0.12)
Low WBC	4.49	(3.49, 5.78)
High SBP	1.28	(1.13, 1.44)
Low SBP	0.74	(0.53, 1.04)
High DBP	1.00	(0.87, 1.15)
Low DBP	0.98	(0.84, 1.14)
High Breath	0.90	(0.80, 1.02)
Low Breath	0.62	(0.04, 9.92)
High Pulse	0.86	(0.77, 0.96)
Low Pulse	0.99	(0.60, 1.63)
GCS_Abnormal	0.40	(0.29, 0.55)
Heart Disease	1.04	(0.87, 1.25)
CVA	0.82	(0.64, 1.05)
CKD	0.99	(0.86, 1.14)
Severe Liver Disease	0.89	(0.73, 1.08)
DM	1.02	(0.88, 1.18)
Hypertension	1.12	(0.97, 1.29)
Cancer	0.85	(0.74, 0.99)

SBP: Systolic Blood Pressure**; DBP:** Diastolic Blood Pressure, **WBC:** White Blood Cells**; GCS:** Glasgow Coma Scale, **CVA:** cerebral vascular accident; **CKD:** Chronic Kidney Disease, **DM:** Diabetes Mellitus
