# Supplementary material for: Comparing machine learning with case-control models to identify confirmed dengue cases
Source: PLoS Negl Trop Dis. 2020 Nov 10;14(11):e0008843. doi: 10.1371/journal.pntd.0008843 (PMC7654779; doi:10.1371/journal.pntd.0008843)
Supplement: S4 Table — (PDF) [file pntd.0008843.s007.pdf]

S4 Table. The software packages employed to build the prediction models and the main characteristics of the DNN model.

Prediction Models	Details in computer software packages adopted
**Decision Tree (DT)**	The rpart package in R (Recursive Partitioning and Regression Trees) with the following parameter settings:<br>split=GINI<br>minbucket=20<br>xval=10<br>cp=0.01
**Deep Neural Networks (DNN)**	Python pytorch, DenseNet architecture with 3 layer and hidden dimension=16
**Logistic Regression (LR)**	Python sklearn's linear model wih solver='lbfgs' and default parameters
