# Supplementary material for: Comparing machine learning with case-control models to identify confirmed dengue cases
Source: PLoS Negl Trop Dis. 2020 Nov 10;14(11):e0008843. doi: 10.1371/journal.pntd.0008843 (PMC7654779; doi:10.1371/journal.pntd.0008843)
Supplement: S5 Table — CVA: cerebral vascular accident; CKD: Chronic Kidney Disease, DM: Diabetes Mellitus. (PDF) [file pntd.0008843.s008.pdf]

S5 Table. Summary of sensitivities, specificities, Positive Prediction Values (PPVs), and accuracies on subgroup analyses with the three prediction models [Decision Tree (DT), Deep Neural Network (DNN) and Logistic Regression (LR)]

	Count	Sensitivity			Specificity			PPV			Accuracy		
		DT	DNN	LR	DT	DNN	LR	DT	DNN	LR	DT	DNN	LR
**Overall**	4894	90.30%	90.00%	90.00%	63.10%	67.40%	64.30%	78.70%	80.60%	79.10%	79.40%	81.00%	79.70%
**Age (years)**													
Age < 18	357	95.50%	86.10%	88.70%	54.80%	75.80%	71.90%	66.80%	77.20%	74.20%	74.60%	80.80%	79.90%
18 ≤ age < 65	3253	91.40%	89.90%	89.10%	65.30%	68.80%	68.10%	78.10%	79.60%	79.10%	80.30%	80.90%	80.20%
65 ≤ age	1284	87%	91.10%	92.10%	59.40%	58.70%	46.40%	83.20%	83.60%	79.90%	78.60%	81.30%	78.40%
**Gender**													
Female	2418	91.50%	92.20%	92.60%	61.50%	65.20%	62.60%	78.80%	80.50%	79.40%	79.80%	81.60%	80.90%
Male	2476	89%	87.80%	87.40%	64.60%	69.50%	65.80%	78.60%	80.80%	78.80%	79.10%	80.40%	78.60%
**Epidemic periods**													
Pre-peak: wks ≤ 35	549	92.60%	92.80%	93.10%	53.60%	59.10%	54.20%	84.10%	85.80%	84.40%	81.90%	83.60%	82.40%
Peak: 35 <wks ≤ 40	2989	91.20%	90.80%	90.50%	64%	68.40%	65.90%	81.80%	83.60%	82.50%	81.40%	82.70%	81.60%
Post-peak: 40 < wks	1356	86.10%	85.90%	86.50%	63.80%	67.70%	63.90%	67.40%	69.80%	67.60%	74.10%	76.20%	74.40%
**Body Temp (°C)**													
Temp >= 38	3051	93.60%	92.40%	91.70%	52.70%	58.20%	57.10%	79.30%	81.10%	80.60%	79.70%	80.80%	80.00%
Temp < 38	1843	83%	84.70%	86.20%	75%	77.90%	72.30%	77.10%	79.50%	76.00%	79%	81.30%	79.30%
**White Blood Cells**													
Low	743	99.60%	99.70%	99.90%	0.70%	2.00%	0.00%	89.10%	89.30%	89.10%	88.80%	89.00%	89.00%

Normal	3327	92.10%	90.70%	91.20%	46.20%	54.00%	49.40%	75.90%	78.40%	76.80%	75.90%	77.80%	76.50%
High	824	9%	26.00%	16.90%	98.60%	97.40%	96.60%	53.20%	64.70%	46.70%	85.10%	86.70%	84.60%
**Platelets**													
Low	746	94%	97.10%	95.20%	21.10%	30.30%	23.60%	85.90%	87.50%	86.20%	82%	86.00%	83.30%
Normal	4148	89.20%	88.10%	88.60%	66%	69.90%	67.00%	76.80%	78.80%	77.30%	79%	80.10%	79.10%
**Comorbidities**													
Heart Disease	545	89.60%	90.30%	90.00%	61.90%	64.90%	59.60%	78.60%	80.00%	77.60%	78.80%	80.40%	78.10%
CVA	265	88%	90.00%	88.90%	62.50%	66.30%	65.50%	74.50%	76.90%	76.20%	76.60%	79.40%	78.50%
CKD	1089	87.20%	89.00%	87.90%	63.30%	65.90%	57.80%	78.10%	79.60%	75.70%	77.60%	79.70%	75.90%
Severe Liver Disease	435	90.90%	90.80%	91.40%	56.70%	62.20%	57.10%	73.90%	76.50%	74.20%	76.30%	78.70%	76.80%
DM	880	89.60%	89.80%	90.00%	64.10%	66.70%	64.90%	79.20%	80.50%	79.70%	79.50%	80.70%	80.10%
Hypertension	938	91.70%	92.30%	92.40%	59.30%	61.80%	57.90%	78.80%	80.00%	78.40%	79.50%	80.80%	79.40%
Cancer	926	90.90%	90.60%	92.90%	58.10%	62.60%	60.80%	74.10%	76.20%	75.80%	76.80%	78.60%	79.10%

CVA: cerebral vascular accident; **CKD:** Chronic Kidney Disease, **DM:** Diabetes Mellitus
