# Supplementary material for: Comparing machine learning with case-control models to identify confirmed dengue cases
Source: PLoS Negl Trop Dis. 2020 Nov 10;14(11):e0008843. doi: 10.1371/journal.pntd.0008843 (PMC7654779; doi:10.1371/journal.pntd.0008843)
Supplement: S6 Table — CVA: cerebral vascular accident; CKD: Chronic Kidney Disease, DM: Diabetes Mellitus. (PDF) [file pntd.0008843.s009.pdf]

S6 Table. **Subgroup analysis in the Decision Tree (DT) Model**

DT	Count	Sensitivity	Specificity	PPV	Accuracy
**Overall**	4894	90.30%	63.10%	78.70%	79.40%
		89.7%-90.9%	62.2%-64.1%	78.2%-79.1%	79%-79.9%
**Age (years)**					
Age < 18	357	95.50%	54.80%	66.80%	74.60%
		93.4%-97.6%	52.2%-57.4%	65.4%-68.1%	73%-76.2%
18 ≤ age < 65	3253	91.40%	65.30%	78.10%	80.30%
		90.6%-92.1%	64.2%-66.4%	77.5%-78.6%	79.7%-80.9%
65 ≤ age	1284	87%	59.40%	83.20%	78.60%
		86%-87.9%	57.3%-61.4%	82.5%-83.9%	77.8%-79.5%
**Gender**					
Female	2418	91.50%	61.50%	78.80%	79.80%
		90.6%-92.5%	60.4%-62.7%	78.2%-79.3%	79%-80.6%
Male	2476	89%	64.60%	78.60%	79.10%
		88.4%-89.6%	63.4%-65.9%	78.1%-79.1%	78.7%-79.5%
**Epidemic periods**					
Pre-peak: wks ≤ 35	549	92.60%	53.60%	84.10%	81.90%
		91.6%-93.6%	51.4%-55.7%	83.5%-84.8%	81%-82.9%
Peak: 35 < wks ≤ 40	2989	91.20%	64%	81.80%	81.40%
		90.5%-91.8%	62.6%-65.5%	81.2%-82.4%	80.8%-82%
Post-peak: 40 < wks	1356	86.10%	63.80%	67.40%	74.10%
		85.1%-87%	62.7%-64.8%	66.9%-67.9%	73.6%-74.6%
**Body Temp (°C)**					
Temp >= 38	3051	93.60%	52.70%	79.30%	79.70%
		92.8%-94.4%	51.4%-53.9%	78.9%-79.7%	79.2%-80.2%
Temp < 38	1843	83%	75%	77.10%	79%
		82.1%-83.9%	72.9%-77.2%	75.6%-78.6%	77.9%-80.1%
**White Blood Cells**					
Low	743	99.60%	0.70%	89.10%	88.80%
		98.9%-100%	0%-3.0%	89.0%-89.3%	88.3%-89.4%
Normal	3327	92.10%	46.20%	75.90%	75.90%
		91.3%-92.8%	44.6%-47.9%	75.4%-76.5%	75.3%-76.6%
High	824	9%	98.60%	53.20%	85.10%
		4.4%-13.6%	98%-99.2%	33.3%-73.1%	84.2%-86.1%
**Platelets**					
Low	746	94%	21.10%	85.90%	82%
		92.9%-95%	18.6%-23.5%	85.5%-86.2%	81.2%-82.8%
Normal	4148	89.20%	66%	76.80%	79%
		88.5%-90%	64.9%-67.1%	76.3%-77.4%	78.4%-79.5%
**Comorbidities**					

Heart Disease	545	89.60%	61.90%	78.60%	78.80%
		88.4%-90.8%	60.4%-63.5%	77.9%-79.3%	77.9%-79.7%
CVA	265	88%	62.50%	74.50%	76.60%
		85.6%-90.3%	58.9%-66.1%	72.7%-76.3%	74.7%-78.5%
CKD	1089	87.20%	63.30%	78.10%	77.60%
		86.3%-88%	62.1%-64.5%	77.4%-78.7%	76.8%-78.4%
Severe Liver Disease	435	90.90%	56.70%	73.90%	76.30%
		89.3%-92.5%	54.1%-59.2%	72.8%-75.1%	75%-77.7%
DM	880	89.60%	64.10%	79.20%	79.50%
		88.5%-90.6%	62.3%-65.9%	78.5%-80%	78.8%-80.2%
Hypertension	938	91.70%	59.30%	78.80%	79.50%
		90.6%-92.8%	57.4%-61.2%	78%-79.6%	78.5%-80.4%
Cancer	926	90.90%	58.10%	74.10%	76.80%
		89.5%-92.4%	56.3%-59.9%	73.2%-75%	75.6%-77.9%

CVA: cerebral vascular accident; **CKD:** Chronic Kidney Disease, **DM:** Diabetes Mellitus
