# Supplementary material for: Comparing machine learning with case-control models to identify confirmed dengue cases
Source: PLoS Negl Trop Dis. 2020 Nov 10;14(11):e0008843. doi: 10.1371/journal.pntd.0008843 (PMC7654779; doi:10.1371/journal.pntd.0008843)
Supplement: S7 Table — CVA: cerebral vascular accident; CKD: Chronic Kidney Disease, DM: Diabetes Mellitus. (PDF) [file pntd.0008843.s010.pdf]

S7 Table. Subgroup analysis in the Deep Neural Network (DNN) Model

DNN	Count	Sensitivity	Specificity	PPV	Accuracy
**Overall**	4894	90.00%	67.40%	80.60%	81.00%
		89.9%-90.1%	66.1%-68.7%	80.0%-81.3%	80.4%-81.5%
**Age (years)**					
Age < 18	357	86.10%	75.80%	77.20%	80.80%
		82.5%-89.6%	72.0%-79.6%	74.5%-79.9%	78.4%-83.2%
18 ≤ age < 65	3253	89.90%	68.80%	79.60%	80.90%
		89.3%-90.4%	67.1%-70.4%	78.7%-80.4%	80.1%-81.7%
65 ≤ age	1284	91.10%	58.70%	83.60%	81.30%
		90.0%-92.2%	56.1%-61.3%	82.8%-84.5%	80.3%-82.4%
**Gender**					
Female	2418	92.20%	65.20%	80.50%	81.60%
		91.5%-92.8%	63.4%-67.0%	79.7%-81.3%	80.8%-82.4%
Male	2476	87.80%	69.50%	80.80%	80.40%
		87.2%-88.4%	68.2%-70.8%	80.1%-81.5%	79.7%-81.1%
**Epidemic periods**					
Pre-peak: wks ≤ 35	549	92.80%	59.10%	85.80%	83.60%
		91.7%-93.9%	56.1%-62.0%	84.9%-86.7%	82.5%-84.7%
Peak: 35 < wks ≤ 40	2989	90.80%	68.40%	83.60%	82.70%
		90.3%-91.2%	67.2%-69.6%	83.1%-84.1%	82.1%-83.3%
Post-peak: 40 < wks	1356	85.90%	67.70%	69.80%	76.20%
		84.6%-87.2%	65.4%-70.0%	68.3%-71.3%	74.9%-77.5%
**Body Temp (°C)**					
Temp >= 38	3051	92.40%	58.20%	81.10%	80.80%
		92.1%-92.8%	56.5%-59.8%	80.5%-81.7%	80.2%-81.4%
Temp < 38	1843	84.70%	77.90%	79.50%	81.30%
		83.9%-85.5%	76.3%-79.5%	78.4%-80.7%	80.5%-82.2%
**White Blood Cells**					
Low	743	99.70%	2.00%	89.30%	89.00%
		99.3%-100.1%	-0.054	89.0%-89.5%	88.6%-89.4%
Normal	3327	90.70%	54.00%	78.40%	77.80%
		90.4%-91.0%	52.2%-55.8%	77.7%-79.1%	77.1%-78.4%
High	824	26.00%	97.40%	64.70%	86.70%
		22.5%-29.5%	96.2%-98.7%	53.5%-75.9%	85.6%-87.8%

**Platelets**					
Low	746	97.10%	30.30%	87.50%	86.00%
		96.3%-97.8%	26.6%-34.0%	86.9%-88.1%	85.1%-86.9%
Normal	4148	88.10%	69.90%	78.80%	80.10%
		87.9%-88.3%	68.5%-71.4%	78.0%-79.6%	79.4%-80.8%
**Comorbidities**					
Heart Disease	545	90.30%	64.90%	80.00%	80.40%
		89.1%-91.5%	63.3%-66.5%	79.3%-80.8%	79.4%-81.3%
CVA	265	90.00%	66.30%	76.90%	79.40%
		87.9%-92.0%	62.2%-70.4%	74.7%-79.1%	77.3%-81.6%
CKD	1089	89.00%	65.90%	79.60%	79.70%
		88.2%-89.8%	63.9%-67.9%	78.6%-80.6%	78.8%-80.7%
Severe Liver Disease	435	90.80%	62.20%	76.50%	78.70%
		88.8%-92.8%	58.3%-66.2%	74.7%-78.2%	77.0%-80.3%
DM	880	89.80%	66.70%	80.50%	80.70%
		89.0%-90.7%	64.3%-69.0%	79.3%-81.6%	79.6%-81.8%
Hypertension	938	92.30%	61.80%	80.00%	80.80%
		91.2%-93.3%	59.7%-63.9%	79.1%-80.8%	79.9%-81.6%
Cancer	972	90.60%	62.60%	76.20%	78.60%
		89.4%-91.9%	59.6%-65.6%	74.8%-77.6%	77.2%-79.9%

CVA: cerebral vascular accident; **CKD:** Chronic Kidney Disease, **DM:** Diabetes Mellitus
