# Supplementary material for: Comparing machine learning with case-control models to identify confirmed dengue cases
Source: PLoS Negl Trop Dis. 2020 Nov 10;14(11):e0008843. doi: 10.1371/journal.pntd.0008843 (PMC7654779; doi:10.1371/journal.pntd.0008843)
Supplement: S8 Table — CVA: cerebral vascular accident; CKD: Chronic Kidney Disease, DM: Diabetes Mellitus. (PDF) [file pntd.0008843.s011.pdf]

S8 Table. Subgroup analysis in the Logistic Regression (LR) Model

LR	Count	Sensitivity	Specificity	PPV	Accuracy
**Overall**	4894	90.00%	64.30%	79.10%	79.70%
		89.9%-90.1%	64.0%-64.5%	79.0%-79.3%	79.6%-79.8%
**Age (years)**					
Age < 18	357	88.70%	71.90%	74.20%	79.90%
		87.5%-89.9%	70.6%-73.2%	73.3%-75.2%	79.0%-80.9%
18 ≤ age < 65	3253	89.10%	68.10%	79.10%	80.20%
		88.9%-89.3%	67.9%-68.3%	79.0%-79.2%	80.0%-80.3%
65 ≤ age	1284	92.10%	46.40%	79.90%	78.40%
		92.0%-92.3%	45.8%-47.0%	79.8%-80.1%	78.2%-78.6%
**Gender**					
Female	2418	92.60%	62.60%	79.40%	80.90%
		92.5%-92.8%	62.2%-63.1%	79.2%-79.6%	80.7%-81.1%
Male	2476	87.40%	65.80%	78.80%	78.60%
		87.2%-87.6%	65.5%-66.1%	78.7%-79.0%	78.5%-78.7%
**Epidemic periods**					
Pre-peak: wks ≤ 35	549	93.10%	54.20%	84.40%	82.40%
		92.6%-93.5%	53.3%-55.1%	84.1%-84.7%	82.0%-82.9%
Peak: 35 < wks ≤ 40	2989	90.50%	65.90%	82.50%	81.60%
		90.4%-90.6%	65.6%-66.2%	82.4%-82.6%	81.5%-81.8%
Post-peak: 40 < wks	1356	86.50%	63.90%	67.60%	74.40%
		86.3%-86.8%	63.5%-64.3%	67.3%-67.8%	74.2%-74.7%
**Body Temp (°C)**					
Temp >= 38	3051	91.70%	57.10%	80.60%	80.00%
		91.6%-91.9%	56.8%-57.5%	80.5%-80.7%	79.8%-80.1%
Temp < 38	1843	86.20%	72.30%	76.00%	79.30%
		86.0%-86.5%	72.0%-72.6%	75.8%-76.1%	79.2%-79.5%
**White Blood Cells**					
Low	743	99.90%	0.00%	89.10%	89.00%
		99.8%-100.0%	0.0%-0.0%	89.1%-89.1%	88.9%-89.1%
Normal	3327	91.20%	49.40%	76.80%	76.50%
		91.1%-91.3%	49.0%-49.8%	76.7%-77.0%	76.3%-76.6%
High	824	16.90%	96.60%	46.70%	84.60%
		16.3%-17.5%	96.4%-96.8%	45.0%-48.5%	84.4%-84.8%
**Platelets**					
Low	746	95.20%	23.60%	86.20%	83.30%
		94.9%-95.5%	22.3%-24.9%	86.0%-86.4%	82.9%-83.6%
Normal	4148	88.60%	67.00%	77.30%	79.10%
		88.5%-88.7%	66.7%-67.3%	77.2%-77.5%	79.0%-79.2%
**Comorbidities**					
Heart Disease	545	90.00%	59.60%	77.60%	78.10%

		89.3%-90.6%	59.2%-60.0%	77.4%-77.8%	77.7%-78.5%
CVA	265	88.90%	65.50%	76.20%	78.50%
		87.8%-90.0%	64.5%-66.4%	75.8%-76.7%	77.9%-79.0%
CKD	1089	87.90%	57.80%	75.70%	75.90%
		87.6%-88.2%	57.3%-58.3%	75.5%-75.9%	75.6%-76.1%
Severe Liver Disease	435	91.40%	57.10%	74.20%	76.80%
		91.0%-91.8%	55.8%-58.4%	73.6%-74.8%	76.2%-77.5%
DM	880	90.00%	64.90%	79.70%	80.10%
		89.7%-90.3%	64.5%-65.4%	79.5%-79.9%	79.8%-80.4%
Hypertension	938	92.40%	57.90%	78.40%	79.40%
		92.0%-92.8%	57.4%-58.4%	78.2%-78.6%	79.1%-79.7%
Cancer	926	92.90%	60.80%	75.80%	79.10%
		92.7%-93.2%	60.3%-61.4%	75.5%-76.0%	78.8%-79.3%

CVA: cerebral vascular accident; **CKD:** Chronic Kidney Disease, **DM:** Diabetes Mellitus
