# Supplementary material for: Comparing machine learning with case-control models to identify confirmed dengue cases
Source: PLoS Negl Trop Dis. 2020 Nov 10;14(11):e0008843. doi: 10.1371/journal.pntd.0008843 (PMC7654779; doi:10.1371/journal.pntd.0008843)
Supplement: Attachment — Submitted filename: PNTD-D-20-00034-Response to Reviewers-07210810-checked.pdf [file pntd.0008843.s012.pdf]

Associate Editor Comments

1) The Discussion should be substantially shorter and more focused. As a particular example, the discussion about pathogenesis of leukopenia and thrombocytopenia is unnecessary since these findings are well described and the current study provides no new insights into pathogenesis.

[Response]

We appreciate this crucial comment and revised the section **Discussion** accordingly. The word counts of the **Discussion** were 578 shorter (from 3265 to 2687) than the original manuscript. **[Page 21-29]**

2) The analysis of previous literature is incomplete. The authors need to cite previous papers on prediction models and compare them to the current results. Specific papers not addressed include: Tanner et al. PLoS Negl Trop Dis 2008, Potts et al. PLoS Negl Trop Dis 2010, Park et al. PLoS Negl Trop Dis 2018, and Phakhounthong et al. BMC Pediatr 2018.

[Response]

We have addressed this issue by incorporating a new paragraph in section **Introduction** that reads "We addressed this challenge by exploiting machine learning based approaches and …but it is almost impossible for a user to figure out how a prediction is made" **[Page 5, Lines 128-141]**

3) The authors' analysis was limited for obvious reasons to DLI, which, as defined, required the clinician's suspicion of arboviral disease. This represents a potentially significant source of bias, of particular relevance to use of the algorithm for real-time syndromic surveillance as proposed by the authors. The authors should at a minimum acknowledge this limitation and discuss the potential effects of this bias, especially for the comparison of sensitivity and specificity during different time periods. Can the authors address how many cases in the full dataset (>100K visits) during each month would potentially have been identified as suspected dengue by these algorithms?

[Response]

We have incorporated a new subsection "**Analyses of the excluded cases**" in section **Results**. This subsection reads "It was of interest to learn how our prediction models performed with the excluded cases. …In fact, as shown in the S5 table, among these 1,100 cases, the numbers of cases for which the predictions made by our models matched physicians' diagnoses were consistently around 900." **[Page 17, Lines 355-373]**

4) Table S1 shows statistically significant differences between included and excluded groups. The authors should address the potential implications of these differences for the results. Did the authors review whether these subjects could be included in validation of the final models?

[Response]

We have added the following paragraph in the section **Discussion** to address the potential implications of these differences. "Fifth, we conjecture that those thrombocytopenia cases excluded from our study population should be dengue cases and should have dengue confirmatory test before visiting NCKUH ED…If these cases were included in the final models, the positive predictive values of our models could be improved." **[Pages 27, Lines 592-597]** We also provide the simulation results in the **S4 Fig [Page 40-41]**, which is in line with our hypothesis.

5) Line 188- Why were these cutoffs selected? Would other cutoff values have had greater discriminatory value?

[Response]

The cutoff values represented the reference (i.e. normal ranges) of the tests which have been routinely used in the NCKU hospital with greater discrimination. We then employed these cutoff values, representing which represented reference ranges, shown in **S2 Table [Page 43]**, to stratify as "normal" vs "abnormal" (high or low) values these variables and computed the crude odds ratios of the 18 variables.

6) The WHO definitions (noted in Figure 3) should be described in Methods.

[Response]

We have described the WHO definitions in section **Methods** as below. "The clinical diagnosis of dengue-like illness in Taiwan was usually made according to the 1997 or 2009 WHO clinical definitions … The reported sensitivity and specificity of the 1997 and 2009 WHO definitions in predicting dengue [26] were also presented in the Figure 3 for better comparison." **[Pages 14-15, Lines 307-317]**

7) Table 3- The clinical relevance of showing results for subgroups based on laboratory data included in the models (e.g., WBC, platelet) is unclear. These subgroup analyses should be deleted.

[Response]

Leukopenia (low White Blood Cell (WBC)) has been widely used for global clinicians and the WHO as first laboratory clue of clinical suspicion of dengue disease. Rapidly decreasing platelet counts was also listed as warning signs in 2009 WHO classification. Therefore the accuracy of clinical diagnosis of dengue will be affected by these

laboratory results. We moved the Table 3 “Summary of sensitivities, specificities, Positive Prediction Values (PPVs), and accuracies on subgroup analyses with the three prediction models [Decision Tree (DT), Deep Neuro Network (DNN) and Logistic Regression (LR)] to the supplementary **S7 Table** [**Pages 48-49**] which shows the data that the reviewers asked sensitivities and specificities at different epidemic periods more clearly.

8) Do the authors have data on day of illness at presentation? If so, these data should be included in Table 1. Did they consider including this variable in the analysis?

[Response]

We have added the following paragraph in the section **Discussion** to address this important issue. “Since we did not don’t have data on day of illness (fever day) at presentation, it’s impossible to know which phases of dengue natural course the patient is at…We are trying to include this information into the entry in our electronic medical record (EMR) system in the near future.” **[Pages 27-28, Lines 599-608]**

9) The timing of the epidemic in Taiwan in 2015 should be noted under Study Population.

[Response]

The timing of the epidemic in Taiwan in 2015 have been noted in subsection “**Study Population**” as following. “An unprecedented dengue epidemic occurred in Taiwan during 2015 and resulted in 22,777 laboratory-confirmed cases [30]. S1 Fig shows the epidemic curve.” **[Pages 6, Lines 159-161]**

10) Line 147- The authors should note the number of visits excluded for incomplete records, cancellation, or re-admission.

[Response]

We have discussed the numbers of cases excluded due to cancellation and re-admission in the following paragraph in subsection “**Study population**” of section **Methods**. “In total, there were 100,491 visits to the ED of NCKUH (NCKUH-ED) during 2015. Among them, 3698 patients canceled the emergency consultation and therefore were excluded… In other words, the numbers of excluded cases and merged cases were not affected by the dengue endemic.” **[Page 7, Lines 170-176]**

As shown in the following table, the numbers of cases excluded due to cancellation and re-admission essentially stayed steady throughout the entire year.

**Year-Month**	**re-admission**	**cancellation**
2015-01	543	305

2015-02	503	281
2015-03	531	315
2015-04	547	291
2015-05	575	311
2015-06	531	297
2015-07	482	273
2015-08	590	299
2015-09	622	397
2015-10	609	307
2015-11	515	311
2015-12	563	311
**Total**	**6611**	**3698**

11) Lines 298-302- It is unnecessary to repeat data presented in Table 1 in the text.
[Response]
All of the repeated text was deleted, according to your kind suggestions.

12) Figure S1 and S2- The labels on each branch should be reformatted to avoid overwriting the graphical representation of the decision tree.
[Response]
Figures S1 and S2 (**S2 and S3** in the revised manuscript) were reformatted to avoid overwriting the graphical representation of the decision tree. **[Pages 38-39]**

13) References 54 and 57 are identical.
[Response]
Reference 57 was removed accordingly.

Reviewer's comments
[Methods]

Reviewer #1:

1) Dengue is a major health problem worldwide over the past 50 years. The challenge of identifying dengue cases is increased during large epidemics, especially in non-endemic areas with limited experienced staff. The study group used machine learning (ML)-based prediction models to identify dengue confirmed cases with the rationale of applying these models in health facilities where dengue confirmed tests are not available. It is obvious that the objectives of the study clearly articulated with a clear testable hypothesis stated, and the study design is appropriate to address the stated objectives. Study population consisted of dengue-like illness (DLI)

cases (based on ICD9) admitted to the emergency department (ED) from January 1 to December 31, 2015 at National Cheng Kung University Hospital (NCKUH) in Tainan City. The final dataset included 2,942(60.12%) laboratory-confirmed dengue cases and 1,952 non-dengue control cases. The population is clearly described and appropriate for the hypothesis being tested. However, if possible, the authors should present the definition of DLI rather than ICD code.

[Response]

We have presented the definition of DLI in the subsection **"Study Population"** of section **Methods** as follows. "All the clinical diagnoses of DLI were made by clinicians according to the 1997 or 2009 WHO clinical definition of probable dengue [9]. By these definitions, a patient was diagnosed to suffer DLI and coded with corresponding ICD codes, if the patient had fever along with any two of the following clinical features: nausea/vomiting, rash, aches and pains, tourniquet test positive or any warning signs." **[Page 6, Lines 165-169]**

2) The sample size (totally, 4,894 DLI cases) is sufficient to ensure adequate power to address the hypothesis being tested. However, there is an imbalance of cases between the two groups (confirmed dengue cases and non-dengue control cases) which need to be taken into account when interpreting the results from deep neural network (DNN) models, but it was not mentioned in the manuscript. Moreover, some other questions should be concerned: Is there any repeated variable in data set? And what is the total number of samples (rows) in the data set?

[Response]

(1) The dataset contained 2,942(~60%) positive subjects and 1,952(~40%) negative subjects. Since the dataset was not highly unbalanced, we did not employ any procedure to address this issue. In this respect, we have added the following paragraph in subsection "**Prediction models**" of section **Methods** as below. "The last issue with respect to building the prediction models was how the distributions of the dataset should be handled…This issue is of concern only if the numbers of subjects in different groups, e.g. positive and negative, are highly unbalanced." **[Page 12, lines 258-263]**

(2) There was no repeated variable in data set. The total number of samples in the dataset was 4894.

3) Correct statistical analysis was used to support conclusion, and there are no concerns about ethical or regulatory requirements being met. About data validation, internal validation with 2 time-repeats was used. As a suggestion, whether it is better if the models are validated with new data set (external validation)?

[Response]

We have not had large-scale outbreaks in Taiwan to do external validation using the data in different years since 2016 because the annual numbers of indigenous lab.-confirmed dengue cases from 2016 as of May 16, 2020 were 10, 0, 1, 31, 0 in Tainan, respectively. Therefore, we are planning to start international collaboration with those S. E. Asia countries for external validity in the future.

Reviewer #2:

1) Objectives were clearly formulated, the study design is appropriate.

[Response]

Thank you very much for the kind encouragement.

[Results]

Reviewer #1

1) For the results, the analysis presented matches the analysis plan, and the results are clearly and completely presented. The key findings of the study are that using just four input variables [age, body temperature, and counts of white blood cells (WBCs) and platelets], areas under curves (AUCs) of the receiver operating characteristic (ROC) curves were reported at high level (above 80%) for all models, and DNN had higher performance than others. Subgroup analyses were informative, all the models were very sensitive particularly in pre-epidemic period. Pre-peak sensitivity (<35 weeks) was 92.6%, 92.9%, and 93.1% in DT, DNN, and LR respectively. The figures (tables, images) in the manuscript are of sufficient quality for clarity. It is easier for readers to follow up the paper, S1 Fig and S2 Figure should additionally be explained in text. One point should be considered, the algorithms of the highest performance models were not reported. Could the authors provide them in their manuscript?

[Response]

We have inserted the following footnotes into Figures S1 and S2 (**S2 and S3** in the revised manuscript). “This particular tree produced 90.1% sensitivity but only 63.6% specificity.. … Otherwise, the node is colored by blue.” **[Pages 38-39]** Since a DNN model involves multiple layers of non-linear transformation, as addressed in the article entitled “Machine learning in population health: opportunities and threats” published in PLOS Medicine, it is almost impossible for a user to figure out how a prediction is made. Therefore, we will not be able to provide the algorithm of the DNN model. In this respect, we have revised a paragraph in subsection “**Prediction models**” of the section **Methods [Pages 11-12, lines 243-248]** and a paragraph in section **Discussions**. **[Page 23, Lines 496-500]**.

Reviewer #2

1) Results are well presented.

[Response]

Thank you very much for the kind encouragement.

[Conclusions]

Reviewer #1

1) The authors' conclusions are supported by the data presented, and all limitations of the study were clearly reported. Discussion of the manuscript is interesting, and the authors discussed how these data can be helpful to advance our understanding of the topic under study. Results of the study reveal that machine-learning based models can be developed to identify dengue cases with four commonly available key features. This implies that the prediction models can be widely deployed to all levels of medical facilities, including hospitals and local clinics. It would be clearer if the authors emphasize more on how these models will be applied in practice.

[Response]

For practical application of our models in other countries/areas, there are dimensions in the planning. First, it is very important to provide training for collecting consistent variables and data for all levels of medical facilities**,** including hospitals and local clinics. For the primary health care facilities, we had an idea using poster to list all the important variables to be collected. Since most dengue-endemic or hyper-endemic countries have mainly pediatric cases, other important variables can also be included. If researchers are interested in models for severe dengue cases, other variables such as comorbidities of diabetes etc. can also be collected. In conclusion, area adjustment using several different local data sets to overcome the weaknesses of a certain data set is necessary. [See **Page 26**, **Lines 566-569** of the section **Discussion**].

The second dimension has to consider computer facilities that we wrote into different parts of this manuscript. For example, a local community hospital/clinic with an instrument of complete blood counts (including platelets) can provide a sentinel screening during outbreaks. Epidemic sites with adequate computer facilities containing a graphic processing unit (GPU) can carry out the training efficiently, the DNN models can be applied to achieve the highest prediction performance. In contrast, at sites with very limited or even no computer facilities, the DT models or the explicit

prediction logic regression model alone can be used with a typical personal computers or lap-tops to obtain reasonable prediction performance. Once the training process is completed, the DNN model can be executed on a typical personal computer efficiently. Alternatively, the training process of a DNN model can be executed in a centralized computer facility and then the model can be distributed to local clinics equipped with minimal computer hardware. In other words, once the prediction model is built at central lab, we may still utilize the models in remote areas, with efficient execution of the prediction software. In conclusion, the machine learning approach can facilitate medical and public health efforts to minimize the health threat of dengue epidemics **[**See **Lines 71-73** in the section **Abstract**, **Lines 91-94** in the section **Author Summary**; **Pages 21-22, Lines 432-447, Page 22, Lines 454-458, and Page 24, Lines 517-522** in the section **Discussion]**.

<u>Reviewer #2</u>

1) The authors extract a small number of parameters, the combination of which they conclude are predictive of dengue during an outbreak in Taiwan. The identified parameters would also hold true for COVID-19: lymphopenia, fever, etc. In fact, during SARS in Singapore in 2003, overlapping parameters were found for dengue and SARS: Clin Infect Dis. 2004 Dec 15;39(12):1818-23. Epub 2004 Nov 19. Use of simple laboratory features to distinguish the early stage of severe acute respiratory syndrome from dengue fever. The authors should highlight that laboratory confirmation remains the primary goal of surveillance and outbreak investigation. Artificial intelligence and other parameters may aid when laboratories are overwhelmed, but should never replace laboratory confirmation. in fact, the call is for more enhanced laboratory dengue surveillance in all countries, including low to middle income countries.

[Response]

We have added a paragraph in the section **Discussion** to address the COVID-19 challenge and highlight that laboratory confirmation remains the primary goal of surveillance and outbreak investigation. "Finally, dengue and coronavirus disease 2019 (COVID-19) are difficult to distinguish because they share common clinical and laboratory features…However, it should never replace laboratory-confirmation, even in low and middle income countries." **[Page 28, Lines 614-626]**

[Editorial and Data Presentation Modifications]

<u>Reviewer #1</u>

1) The discussion is sometimes rather long for the readers to focus, and should be more concise.

[Response]

The revised section **Discussion** is more concise with focusing on our results and further applications. **[Page 21-29]**

<u>Reviewer #2</u>

1) none

[Response]

Thank you very much.

[Summary and General Comments]

<u>Reviewer #1</u>

The study group used novel machine learning -based prediction models to identify dengue confirmed cases with the rationale of applying these models in health facilities where dengue confirmed tests are not available. As mentioned above, the prediction models to identify dengue cases with four commonly available key features, and can be widely implemented in all levels of medical facilities, and serve as a key component in an integrated dengue surveillance system. Overall, the manuscript is well prepared and organized. I recommend it is considered to be published with minor revision.

[Response]

Thank you for the kind encouragement and we hope this revised manuscript will be acceptable.

<u>Reviewer #2</u>

The authors extract a small number of parameters, the combination of which they conclude are predictive of dengue during an outbreak in Taiwan. The identified parameters would also hold true for COVID-19: lymphopenia, fever, etc. In fact, during SARS in Singapore in 2003, overlapping parameters were found for dengue and SARS: Clin Infect Dis. 2004 Dec 15;39(12):1818-23. Epub 2004 Nov 19. Use of simple laboratory features to distinguish the early stage of severe acute respiratory syndrome from dengue fever. The authors should highlight that laboratory confirmation remains the primary goal of surveillance and outbreak investigation. Artificial intelligence and other parameters may aid when laboratories are overwhelmed, but should never replace laboratory confirmation. in fact, the call is for more enhanced laboratory dengue surveillance in all countries, including

low to middle income countries.

[Response]

We agreed with the reviewer that laboratory surveillance is crucially important and our study using machine learning methods can serve as an assisting role when surging capacity of laboratories have difficulties during large-scale outbreaks. We also include two paragraphs in the section **Discussion** to highlight your insightful comments**:** "Finally, dengue and coronavirus disease 2019 (COVID-19) are difficult to distinguish because they share common clinical and laboratory features…However, it should never replace laboratory-confirmation, even in low and middle income countries." **[Page 28, Lines 614-626]**

[Figure Files]

While revising your submission, please upload your figure files to the Preflight Analysis and Conversion Engine (PACE) digital diagnostic tool, https://pacev2.apexcovantage.com. PACE helps ensure that figures meet PLOS requirements. To use PACE, you must first register as a user. Then, login and navigate to the UPLOAD tab, where you will find detailed instructions on how to use the tool. If you encounter any issues or have any questions when using PACE, please email us at figures@plos.org.

[Response]

Dr. TS Ho representing our study group had registered as a user of the PACE and then double-checked our figure files before final uploading.

[Data Requirements]

Please note that, as a condition of publication, PLOS' data policy requires that you make available all data used to draw the conclusions outlined in your manuscript. Data must be deposited in an appropriate repository, included within the body of the manuscript, or uploaded as supporting information. This includes all numerical values that were used to generate graphs, histograms etc.. For an example see here: http://www.plosbiology.org/article/info%3Adoi%2F10.1371%2Fjournal.pbio.1001908#s5.

[Response]

We will provide one set of raw data to generate main figures and tables.

[Reproducibility]

To enhance the reproducibility of your results, PLOS recommends that you deposit laboratory protocols in protocols.io, where a protocol can be assigned its own identifier

(DOI) such that it can be cited independently in the future. For instructions see https://journals.plos.org/plosntds/s/submission-guidelines#loc-methods

[Response]

We have will deposit the protocols of our established DT, DNN, and LR models. If users have any questions, please contact with us through the E-mail numbers.
