# Supplementary material for: Comparing machine learning with case-control models to identify confirmed dengue cases
Source: PLoS Negl Trop Dis. 2020 Nov 10;14(11):e0008843. doi: 10.1371/journal.pntd.0008843 (PMC7654779; doi:10.1371/journal.pntd.0008843)
Supplement: Attachment — Submitted filename: Responses to Reviewers comments-2020-0924-0019.pdf [file pntd.0008843.s013.pdf]

Point-by-Point Responses to the Editors and Reviewers

Q1

In the previous review, the authors were asked to address potential bias due to the criteria for selection of cases (DLI as defined by ICD-9 codes or request for diagnostic laboratory testing). The authors have provided a detailed tabulation of the number of cases per month, as requested.

Q1-1: *The authors were also requested to "acknowledge this limitation and discuss the potential effects of this bias"; this point was not addressed. The authors need to modify the Discussion to acknowledge the reliance of their study design on clinicians making a diagnosis of DLI and requesting diagnostic testing.*

[Response]

We added a paragraph of potential effects of the bias in the 4th limitation of the Discussion: "Fourth, our selection of 90% sensitivity and 80% specificity may not be suitable in all epidemiological settings. Besides, in the current model, the machine was trained to pick up laboratory-confirmed dengue cases from a specific pool of dengue-like illness, which rely on clinicians making a diagnosis of DLI and requesting diagnostic testing of dengue. Therefore, the sensitivity, specificity, and predictive value of our algorithms depend upon the distribution of other clinical diagnoses in the study population." (Pages 25, Lines 561-567).

Q1-2: *As part of their response, the authors have provided new analyses of excluded cases in the revised manuscript. However, the presentation of these analyses introduces a number of new issues. First, under Results, the section describing their analysis of excluded cases has been placed before the description of the results of the modeling; these sections needs to be re-ordered.*

[Response]

Thanks for your suggestion. We have deleted the section describing the analysis of excluded cases. (Please see the "integrated answers for Q1-2, 1-3 and 1-4" after Q1-4).

Q1-3: *Second, the analyses presented in figure S4 and described in the Discussion (lines 592-597) are problematic because they make an assumption that these are true dengue cases and limit consideration to cases with thrombocytopenia who visited the ED after July 2015. It is unsurprising that these steps improve the "accuracy" of the models, but they introduce additional bias into the results; these analyses should be deleted or at least substantially revised.*

[Response]

We have also deleted Figure S4 and those points in the Discussion as your comments to avoid the confusion for readers. (Please see the “integrated answers for Q1-2, 1-3 and 1-4” after Q1-4).

Q1-4: *Third, given the distribution of these cases in time relative to the dengue epidemic, it is not surprising that the authors found a high percentage of cases predicted to be dengue. In actuality, it is somewhat surprising that so few non-DLI subjects had complete data for the 4 key parameters. The ready availability of this information in the clinical setting is in fact proposed as a strength of the models. Can the authors comment on what data elements were missing from the other cases?*

[Integrated Responses for Q1-2, 1-3 and 1-4]

Since our new analyses of the excluded cases caused additional confusions, we decided to delete all the related discussions as well as Figure S4. Meanwhile, we have revised our manuscript to focus on our primary objective and the clinical implications of the two distinctive findings due to our analyses. Our primary objective was to conduct comprehensive analyses on how the prediction models built with alternative machine learning algorithms and comparing different variable sets performed in identifying the laboratory-confirmed dengue cases among those patients with DLI. The two distinctive findings were:

(1) The prediction models built with only 4 key input variables, including age, body temperature, count of white blood cells (WBCs), and count of platelets (PLTs), were able to deliver the same level of performance as the prediction models built by incorporating additional 14 variables, including gender, hemoglobin level, patient’s triage levels at ED, vital signs, and comorbidities.

(2) The three different types of prediction models investigated in our study basically delivered the same level of performance with the DNN models slightly outperforming the DT models and the LR models.

The limitations of our prediction models and the clinical implications of these two distinctive findings were then elaborated in section “Discussions”. (Pages 20-21, Lines 428-470).

Q2

In the previous review, the authors were asked to "highlight that laboratory confirmation remains the primary goal of surveillance and outbreak investigation". The authors have added a very brief acknowledgement of this issue to the Discussion.

Q2-1: *However, an important limitation of their study is that the sensitivity, specificity, and predictive value of their algorithms depend upon the distribution of other diagnoses in the study population; this should be specifically noted in the Discussion.*

[Response]

We added the suggested good points in the Discussion: "…Besides, in the current model, the machine was trained to pick up laboratory-confirmed dengue cases from a specific pool of dengue-like illness, which rely on clinicians making a diagnosis of DLI and requesting diagnostic testing of dengue. Therefore, the sensitivity, specificity, and predictive value of our algorithms depend upon the distribution of other clinical diagnoses in the study population." (Pages 25, Lines 562-567).

Q2-2: *In addition, a cautionary interpretation note about the need for laboratory confirmation needs to be included in the Abstract and Summary.*

[Response]

Thank you very much for your emphasizing this crucially important point. We had added cautionary interpretation notes in the Abstract (lines 64-65) and the Author Summary (lines 85-87).

Q3

In the previous review, the authors were asked to cite several additional published papers on prediction models for dengue and "compare them to the current results". The authors have cited these papers in the Introduction but have not addressed the need to compare their results to these other papers in the Discussion.

[Response]

We have revised parts of "Introduction" and "Discussions" to address this issue. Basically, all the previous studies focused on how to predict dengue diagnoses, dengue phenotypes, or high risk groups of severe illness or even mortality. In our study, we conducted comprehensive analyses on how the prediction models built with alternative machine learning algorithms and different variable sets performed in identifying the laboratory-confirmed dengue cases among those patients with DLI. In comparison with the results from the previous studies, our analyses led to two distinctive observations.

Firstly, the prediction models built with only 4 key input variables, age, body temperature, count of white blood cells (WBCs), and count of platelets (PLTs), were able to deliver the same level of performance as the prediction models built by incorporating additional 14 variables, including gender, hemoglobin level, patient's triage levels at ED, vital signs, and comorbidities.

Secondly, the three different types of prediction models investigated in our study basically delivered the same level of performance with the DNN models slightly outperforming the DT models and the LR models. The clinical implications of these

two distinctive findings were then elaborated in section “Discussions”. (Pages 20-21, Lines 428-470)

Q4

In the previous review, the authors were asked not to repeat data presented in Table 1 in the text (lines 298-302). The authors indicate that this issue was addressed, but their response does not reflect the revised manuscript (lines 334-337).

[Response]

All the repeated data in text were deleted and revised. (Page 16, Lines 335-339)

Q5

In the previous review, the authors were asked to substantially shorten the Discussion, with specific mention of text discussing mechanisms of leukopenia and thrombocytopenia that are not addressed by the present study. While the Discussion has been reduced in length in this revision, it remains overly long and unfocused; for example, the specific sections noted previously were not deleted. (Line 544-551)

[Response]

Sentences discussing mechanisms of leukopenia and thrombocytopenia in the Discussion were all deleted. (Page 24, Lines 535-536)

Q6

The revised Figures S2 and S3 have misspelled "Negative"; this should be corrected.

[Response]

The error parts in the Figures S2 and S3 were all corrected accordingly.

Figure Files:

While revising your submission, please upload your figure files to the Preflight Analysis and Conversion Engine (PACE) digital diagnostic tool, https://pacev2.apexcovantage.com. PACE helps ensure that figures meet PLOS requirements. To use PACE, you must first register as a user. Then, login and navigate to the UPLOAD tab, where you will find detailed instructions on how to use the tool. If you encounter any issues or have any questions when using PACE, please email us at figures@plos.org.

[Response]

Dr. TS Ho representing our study group had registered as a user of the PACE and then double-checked our figure files before final uploading.

Data Requirements:

Please note that, as a condition of publication, PLOS' data policy requires that you make available all data used to draw the conclusions outlined in your manuscript. Data must be deposited in an appropriate repository, included within the body of the manuscript, or uploaded as supporting information. This includes all numerical values that were used to generate graphs, histograms etc.. For an example see here: http://www.plosbiology.org/article/info%3Adoi%2F10.1371%2Fjournal.pbio.1001908#s5.

[Response]

We will provide one set of raw data to generate main figures and tables.

Reproducibility:

To enhance the reproducibility of your results, PLOS recommends that you deposit laboratory protocols in protocols.io, where a protocol can be assigned its own identifier (DOI) such that it can be cited independently in the future. For instructions see http://journals.plos.org/plosntds/s/submission-guidelines#loc-materials-and-methods

[Response]

We will deposit the protocols of our established DT, DNN, and LR models. If users have any questions, please contact with us through the E-mail numbers.
